# Clinical Analysis of Pediatric Glaucoma in Central China

**DOI:** 10.3389/fmed.2022.874369

**Published:** 2022-04-01

**Authors:** Qian Liu, Changgeng Liu, Haijun Li, Xiaoyuan Yang, Yangzeng Dong, Xiaomei Feng, Wenjun Cheng

**Affiliations:** Henan Eye Institute, Henan Eye Hospital, Henan Provincial People's Hospital, Zhengzhou University People's Hospital, Zhengzhou, China

**Keywords:** pediatric glaucoma, trabeculotomy, juvenile open-angle glaucoma, primary congenital glaucoma, intraocular pressure

## Abstract

**Purpose:**

We aimed to describe the characteristics, epidemiology, management, and outcomes of glaucoma in pediatric patients in central China.

**Methods:**

This study retrospectively analyzed inpatients with pediatric glaucoma at Henan Provincial People's Hospital, Henan Eye Institute, and Henan Eye Hospital between 2017 and 2020.

**Results:**

Overall, 239 cases (276 eyes) of pediatric glaucoma in patients, comprising 87 girls (36.40%) and 152 boys (63.60%) were analyzed. The mean age was 6.65 ± 4.46, and 2.93% of the patients had a family history of glaucoma. Primary congenital glaucoma (PCG) was the most common type of glaucoma, followed by traumatic glaucoma in 8.33% of the patients, which was considered secondary glaucoma. The most common signs and symptoms were elevated intraocular pressure (IOP) and eye pain. Trabeculotomy (Trab) and microcatheter-assisted 360° trabeculotomy (MAT) combined with Trab were the most commonly performed surgeries. The IOP of patients with PCG, juvenile open-angle glaucoma (JOAG), and secondary glaucoma were 15.27 ± 7.48 mmHg, 17.16 ± 10.05, and 18.65 ± 8.55, respectively, at the final follow up. The rate of re-operations in patients with PCG, JOAG, and secondary glaucoma were 9.15%, 6.78%, and 4.69%, respectively. The mean visual acuity of the eyes with PCG, JOAG, and secondary glaucoma was 0.79 ± 0.68, 0.51 ± 0.48, and 0.53 ± 0.50, respectively.

**Conclusion:**

PCG, JOAG, and traumatic glaucoma were the most prevalent subtypes in patients with pediatric glaucoma in central China. Trab and MAT combined with Trab were the most common interventions used in this study. Pediatric amblyopia might require full attention during the entire treatment, especially after glaucoma surgery. Effective preventive measures and more public education on glaucoma prevention and the importance of early diagnosis and treatment is necessary.

## Introduction

Pediatric glaucoma is a group of eye diseases that causes irreversible blindness and affects infants and children. Congenital glaucoma is an important risk factor for visual impairment in children (1). Hence, the burden of pediatric glaucoma may substantially diminish the quality of life in affected individuals (2).

The primary goal of glaucoma treatment is intraocular pressure (IOP) reduction, for which medical treatment is often the first-line treatment. For long-term treatment, surgery may be the definitive option for IOP reduction especially in cases of pediatric glaucoma (2). Various types of new glaucoma surgeries have emerged in recent years and may benefit pediatric glaucoma patients (3, 4). Henan Province is in the middle of China and is a traditional agricultural province. Many patients attend and receive treatment at Henan Eye Hospital, which is the leading domestic eye hospital. Therefore, many patients with glaucoma from remote areas are already in an advanced stage of disease at the time of the first visit and are commonly lost to follow-up because of economic or reasons related to long-distance travel. These circumstances may indicate that the possibility of childhood glaucoma resulting in blindness is higher in this province than in economically developed or coastal areas in China.

Pediatric glaucoma, resulting in blindness, is a serious public health problem in Henan. To date, no large studies have examined the characteristics and outcomes of pediatric glaucoma in central China. Therefore, we analyzed the characteristics of pediatric glaucoma in patients at the Department of Ophthalmology, Henan Eye Hospital, Henan Eye Institute, and Henan Provincial People's Hospital.

## Materials and Methods

We conducted a retrospective analysis of all patients with pediatric glaucoma diagnosed and surgically treated at the Department of Ophthalmology of Henan Eye Hospital, Henan Eye Institute, Henan Provincial People's Hospital between 2017 and 2021. Patients with pediatric glaucoma aged <18 years were included. Age, sex, IOP combined with glaucomatous history, visual acuity (VA), abnormal signs under a slit lamp, medications, surgical options, and outcomes were analyzed. This retrospective study was approved by the Ethics Committee of Henan Eye Hospital and Henan Eye Institute and the Henan Provincial People's Hospital Human Research Ethics Committee [approval number HNEECKY-2022(08)]. This study adhered to the tenets of the Declaration of Helsinki. Informed consent was obtained from all guardians or patients.

The patients with pediatric glaucoma in this study were identified by a qualified glaucoma specialist. Surgical treatments were performed if the patient demonstrated progression of glaucomatous damage or, in the surgeon's opinion, the IOP was at a level that would cause additional damage or primary disease requiring treatment. Here, the IOP was measured using a Tono-Pen tonometer and Goldmann applanation tonometer. Pediatric glaucoma is classified as primary or secondary (5, 6). Primary pediatric glaucoma includes primary congenital glaucoma (PCG) and juvenile open-angle glaucoma (JOAG) (7, 8).

### Statistical Analysis

Data were analyzed using SPSS software (version 19.0.0; IBM Corp., Armonk, NY, USA). Continuous and categorical variables are presented as means ± standard deviations and percentages, respectively.

## Results

### Pediatric Glaucoma Characteristics and Epidemiological Findings

In the 5-year study period, 239 glaucoma cases (276 eyes) comprising 87 girls (36.40%) and 152 boys (63.60%) were analyzed. Their demographic characteristics are listed in Table 1. The mean age was 6.65 ± 4.46 years. Overall, 40 eyes received glaucoma surgery, and 38 eyes received non-glaucoma surgery. Six eyes had previously received cataract extraction. A total of 198 eyes had no history of glaucoma surgery, seven patients had a family history of glaucoma, and five patients had a family history of congenital cataracts (Table 1). Of the 276 eyes, 204 eyes (73.91%) were treated with glaucomatous medications before visiting Henan Eye Hospital. Overall, 48.53% eyes received more than one medication for lowering IOP.

**Table 1:** Pediatric glaucoma patient and ocular characteristics.

**Characteristics**	**Total (*N* = 239 patients and 276 eyes)**
Age (at time of surgery)*	
Mean (standard deviation)	6.65 (4.46)
**Gender**	
Male	63.60% (152)
Female	36.40% (87)
**Number of eyes in study**	
1	202
2	37
**Prior surgery**	
**Glaucoma surgery**	**40**
Trabeculectomy (Trab)	19
Trabeculotomy	2
**Non-penetrating trabeculectomy**	2
Endoscopic cyclophotocoagulation (ECP)	2
Gonioscopy-assisted transluminal trabeculotomy (GATT)	1
Microcatheter-assisted 360° trabeculotomy (MAT)	8
Ahmed glaucoma valve implantation	5
Xen glaucoma implant	1
Non-glaucoma surgery	**38**
Phacoemulsification	6
Penetrating Keratoplasty	2
Suture of ocular trauma	5
Vitrectomy	15
Others	10
**Prior numbers of glaucoma medications**
1	66
2	61
3	31
4	7
Uncertain	39
**Glaucoma family history**	**7**
**Congenital cataract**	5

^*^For the 37 patients with both eyes included in the study, their ages at surgery can differ from 0 to 17 years.

### Different Types of Pediatric Glaucoma

A total of 212 eyes had primary glaucoma, and 64 eyes had secondary glaucoma (Table 2). Among the pediatric eyes, PCG accounted for 55.43%. The most common type of the secondary glaucoma in pediatric patients was trauma-related glaucoma (23 eyes; 8.33% of all the involved eyes). Other common types of secondary glaucoma were silicone oil-related (eyes 13, 4.71%) and lens-related (six eyes, 2.17%). Four eyes had uveitis, anterior segment dysgenesis, steroid-induced glaucoma, and retinoblastomas (1.45%).

**Table 2:** Diagnostic findings in patients with surgical pediatric glaucoma^*^.

**Characteristic**	**N (eyes)**	**(%)**
**Primary**	
Primary congenital glaucoma	153	(55.43%)
Male:Female	103:50
Juvenile open-angle glaucoma	59	(21.38%)
Male:Female	36:23
**Secondary**	
**Lens-related**	
Pseudophakia	3	(1.09%)
Subluxation of lens	2	(0.72%)
Aphakia	1	(0.36%)
**Phacomatoses**	
Sturge-Weber Syndrome	1	(0.36%)
**Uveitis**	
Uveitis (intermediate/anterior)	4	(1.45%)
**Anterior segment dysgenesis**		
Aniridia	3	(1.09%)
Peters anomaly	1	(0.36%)
**Others**	
Traumatic	23	(8.33%)
Silicone oil-related	13	(4.71%)
Steroid-induced	4	(1.45%)
Intraocular tumor	4	(1.45%)
Neovascular Glaucoma	3	(1.09%)
Pigment dispersion syndrome	2	(0.72%)

^*^Data are shown as NO. (%).

### Different Signs and Symptoms of Pediatric Glaucoma

The main signs and symptoms observed in this study are summarized in Table 3. Parental concerns were included in the presentation of signs. The common clinical signs observed by an ophthalmologist included elevated IOP (91 eyes, 32.97%), impaired vision (85 eyes, 30.80%), buphthalmos (19 eyes, 6.88%), leukocoria (15 eyes, 5.43%), and enlargement of the C/D ratio (eight eyes, 2.89%). The most common symptoms included eye pain (11 eyes, 3.99%), red eyes (10 eyes, 3.62%), photophobia (nine eyes, 3.26%), lacrimation (seven eyes, 2.54%).

**Table 3:** Different main symptoms and signs of the pediatric glaucoma patients^*^.

**Characteristic**	**N (number of eyes)**	**(%)**
**Signs**	
Elevated IOP	91	(32.97%)
Impaired vision	85	(30.80%)
Buphthalmos	19	(6.88%)
Leukocoria	15	(5.43%)
Enlargement of C/D ratio	8	(2.89%)
Exophthalmos	3	(1.09%)
Strabismus	2	(0.72%)
Nystagmus	1	(0.37%)
Hyphema	1	(0.37%)
**Symptoms**	
Eye pain	11	(3.99%)
Red eye	10	(3.62%)
Photophobia	9	(3.26%)
Lacrimation	7	(2.54%)
Foreign body sensation	1	(0.37%)
**Systemic association**	
Headache	5	(1.81%)
**Uncertain**	8	(2.89%)

*Intraocular pressure: IOP; C/D: cup/disc ratio*.

^*^Data are show as NO. (%).

### Different Interventions for Pediatric Glaucoma

After evaluation by a glaucoma specialist in our center, the patients received surgery as follows: 23.55% underwent trabeculotomy (Trab), 19.93% underwent microcatheter-assisted 360° trabeculotomy (MAT) combined with Trab, and 14.86% underwent Ahmed glaucoma valve (AGV) implantation (Table 4). Eyes with lens-related glaucoma received intraocular lens (IOL) repositioning, IOL suspension fixation, and IOL extraction. Four retinoblastoma eyes received ophthalmectomy.

**Table 4:** Management of pediatric glaucoma patients^*^.

**Characteristic**	**N (numbers of eyes)**	**(%)**
**Surgical treatment**	
Trabeculectomy (Trab)	65	(23.55%)
Microcatheter-assisted 360°trabeculotomy (MAT) combined Trab	55	(19.93%)
Ahmed glaucoma valve implantation	41	(14.86%)
Non-penetrating trabeculectomy	31	(11.23%)
Phacoemulsification	16	(5.80%)
Trabeculotomy combined Trab	15	(5.43%)
MAT	12	(4.35%)
Trabeculotomy	7	(2.54%)
Phacoemulsification combined Ahmed	6	(2.17%)
Ophthalmectomy	4	(1.45%)
Paracentesis and irrigation for hyphema	5	(1.81%)
Removal of silicone	5	(1.81%)
Xen glaucoma implant	2	(0.73%)
Intraocular lens reposition	2	(0.73%)
Evisceration of the eye	2	(0.73%)
Suspension fixation of intraocular lens	1	(0.36%)
Intraocular lens extraction	1	(0.36%)
Ahmed valve reposition	1	(0.36%)
**Others**	
Endoscopic cyclophotocoagulation (ECP)	5	(1.81%)

^*^Data are shown as NO. (%).

### Follow-Up Findings of Patients With Pediatric Glaucoma

The clinical parameters of the different types of glaucoma are described in Table 5. In all patients, the IOP decreased to 16.33 ± 8.28 mmHg at the final follow-up. PCG manifests at an earlier age than JOAG and secondary pediatric glaucoma. Among 153 eyes with PCG, the IOP decreased from 33.56 ± 10.05 to 15.27 ± 7.48 mmHg at the final follow-up. The IOP of patients with JOAG and secondary glaucoma were 17.16 ± 10.05 and 18.65 ± 8.55, respectively, at the final follow up. The VA of pediatric patients with PCG, JOAG, and secondary glaucoma was determined respectively: 8 (5.23%), 4 (6.78%), and 3 (4.69%) eyes had no light perception at the final follow up; 10 (6.54%), 2 (3.39%), and 2 (3.13%) eyes had light perception; 18 (11.76%), 6 (10.17%), and 11 (17.19%) eyes had perceived hand movement; and 9 (5.88%), 7 (11.86%), and 2 (3.13%) eyes had perceived finger counting. The mean VA of the eyes with PCG, JOAG, and secondary glaucoma was 0.79 ± 0.68, 0.51 ± 0.48, and 0.53 ± 0.50, respectively. The rate of reoperation interventions in patients with PCG was 9.15% (14 eyes), which was higher than those of other pediatric glaucoma types. Among eyes with PCG, one eye received three surgeries, and one eye received four surgeries. The number of glaucoma medications used was similar among the different types of glaucoma.

**Table 5:** Final clinical outcomes among different types of pediatric glaucoma^*^.

	**Primary congenital glaucoma**	**Juvenile open-angle glaucoma**	**Secondary glaucoma**
**Presentation**			
AGE	4.59 ± 3.59	9.86 ± 3.67	8.60 ± 4.39
SEX (Male:Female)	82:49	24:20	46:18
IOP (mmHg)	33.56 ± 10.05	32.59 ± 14.99	33.30 ± 11.56
LogMAR visual acuity	NLP~0, (0.89 ± 0.41)^#^	NLP~0, (0.76 ± 0.50) ^#^	NLP~-0.08 (0.83 ± 0.49)^#^
**Final follow-ups**			
IOP (mmHg)	15.27 ± 7.48	17.16 ± 10.05	18.65 ± 8.55
LogMAR visual acuity	NLP~ 0, (0.79 ± 0.68) ^#^	NLP~0, (0.51 ± 0.48) ^#^	NLP~-0.08 (0.53 ± 0.50)^#^
Re-operation interventions/eye	14 (9.15%)	4 (6.78%)	3 (4.69%)
No. of glaucoma medications	2.05 ± 0.98	2.00 ± 1.95	2.07 ± 0.99

^*^Data are shown as mean ± standard deviation or range.

*^#^mean ± standard is from LogMAR visual acuity more than 1.0 in primary congenital glaucoma*.

## Discussion

Glaucoma is the leading cause of irreversible blindness worldwide. Pediatric glaucoma is characterized by impairment of aqueous outflow, resulting in elevated IOP, which further results in glaucomatous optic neuropathy and eventual blindness (9–11). Severe visual impairment in pediatric glaucoma is commonly observed in underdeveloped and developing countries. It is a major and preventable cause of irreversible visual loss in pediatric patients.

Despite recent advances in surgical and medical treatments, pediatric glaucoma has resulted in irreversibly impaired visual function. In underdeveloped and developing countries and districts, the level of medical care is uneven, especially in remote areas. Henan is an agricultural province with limited economic and medical development compared to the coastal and relatively developed areas in China. Pediatric glaucoma may substantially diminish the quality of life over their entire lifetime, which can be devastating to patients and their families, and even to society. To date, epidemiological investigations on pediatric glaucoma in Henan and its surrounding regions have been insufficient. In this study, we included patients with confirmed pediatric glaucoma who underwent surgery in the ophthalmology department of the Henan Eye Institute.

The average age of the patients in the study by Baig et al. was similar to that in another study conducted in Hong Kong, and patients with JOAG were older (2). Surukrattanaskul et al. reported an earlier presentation age in their study (12). This could be related to the different medical referral systems. The number of male pediatric patients was higher than that of female patients (63.60% vs. 36.40%; 1.75:1), which is similar to previously published results (12–16). Moreover, 8.79% of patients in this study had a family history of glaucoma, which was slightly higher than in studies conducted in eastern China and parts of Asia, including Thailand (12, 13). Pediatric glaucoma is commonly treated surgically, and medical treatment is assisted by therapy (17). Here, 16.74% of the patients had previously received at least once glaucoma surgery, 27.62% patients had a history of medical treatment, and 2.93% of the 239 patients with an available family history were identified in this study. A higher rate of glaucoma history was observed in the Dallas Glaucoma Registry (18). The economic development and awareness of prevention could be attributed to this high rate. There was a tendency to check the eyes because of a family history of glaucoma. By contrast, family history was lower in this study than in those reported in Beijing, Shanghai, and Hong Kong districts of China.

PCG was the most prevalent type of glaucoma in this study population, and it was present in 55.43% of the patients. Many studies have reported varied distributions of subtypes of pediatric glaucoma (5, 7, 8, 13, 16, 19). Similar results were observed in mainland reports from Chinese populations of Beijing Tongren Hospital and Shanghai Eye, Ear, Nose and Throat Hospital (13, 14). In the United States, Canada, and Brazil, PCG was the most common diagnosis (7, 15). PCG also comprised the majority of the cases in Britain and the Republic of Ireland (5). Conversely, many studies have reported a higher prevalence of secondary glaucoma than PCG. The prevalence of PCG in Hong Kong and Thailand was similar to the 17% reported in the USA and was much lower than in our studies (12, 16). The number of boys was more than twice that of girls in this study, which is consistent with reports from northern China, the United States, and Europe (20, 21). Papadopoulos et al. reported that the incidence of PCG was significantly greater in pediatric patients of Pakistani origin than in Caucasian children (5). The varied results among different countries and districts suggest that glaucoma subtypes may be related to ethnicity. Although consanguine marriages are not allowed in China, the phenomenon of consanguine marriages might not disappear.

Traumatic glaucoma is the most common type of secondary pediatric glaucoma in Henan Province. Eye trauma is an important cause of ocular morbidity and a leading cause of non-congenital unilateral blindness in children (22). The rate of pediatric traumatic patients was relatively lower in the United States and the United Kingdom (22, 23). In developing countries, the incidence of ocular trauma is higher. Henan Province is an agricultural province and a major source of migrant peasant workers in China. Many young parents go to work without their children. The low level of economic development and child neglect might be some of the reasons for the higher incidence observed in this study compared with previous studies conducted in the Hong Kong district (22, 23). The Tongren Hospital Eye Center has the highest level of specialized care in treating patients with eye trauma, which might lead to a higher incidence of traumatic glaucoma in pediatric patients (14). Therefore, IOP should be measured periodically in pediatric patients after ocular trauma.

Beck et al. have reported that congenital angle anomalies and postoperative inflammation leading to angle dysfunction or synechia closure, and some unknown influences of aphakic state or vitreous interaction may cause reduced aqueous humor outflow (24). Current lens-related glaucoma was less common in this study than that in previous studies. Baig et al. have reported lens-related glaucoma as the most prevalent type of secondary glaucoma (18.0% of all involved eyes) (2). A similar incidence rate was observed (1.6%) on the mainland at Beijing Tongren Hospital. Patients undergoing cataract surgery at an early age are at a high risk of developing glaucoma with or without IOL implantation (25).

Elevated IOP was observed in 32.97% of the patients and was the most common reason for referral and visiting our center. In Hong Kong, only 12.2% of pediatric glaucomatous eyes were identified by high IOP (2). The relatively concentrated medical resources in provincial capital cities in mainland China could be the reason for this. Impaired vision was also an important reason for visiting our center. Medical resources and ophthalmological specialties are quite disparate in mainland China compared with other developed areas worldwide. Hence, most patients with pediatric glaucoma present with an advanced stage of disease and impaired vision at our center. Therefore, early diagnosis and treatment are crucial to prevent vision loss. Moreover, eye pain and redness were the most common symptoms reported by patients or their parents. The results suggest that pediatricians might be familiar with the common symptoms and refer to an ophthalmologist if necessary.

Trab has been effective as the primary approach for pediatric glaucoma (26, 27). Here, the most common initial glaucoma intervention for each eye was Trab. The most commonly performed surgery was Trab and MAT combined with Trab. Thus, Trab remains the preferred surgical treatment for pediatric glaucoma in central China. The most common surgical procedure in North America and the United Kingdom is goniotomy (7, 28, 29). However, Trab and MAT combined with Trab have been adopted rather than goniotomy for anterior angle surgery in China (14). Previous reports have shown that angle surgeries (regardless of goniotomy or Trab) were effective for PCG, JOAG, and uveitic glaucoma (30–36). Non-penetrating trabeculectomy was used to treat the patients with PCG and JOAG. AGV was treated for multiple surgical failures or secondary pediatric glaucoma in this study. In Hong Kong, the most common surgical intervention is AGV (16). Similar to the Hong Kong report, we observed that patients with aniridia accepted AGV as the primary treatment in central China (16). Differences in economic levels and healthcare systems could be one of the influencing factors in the different conditions of pediatric glaucoma treatments.

Here, in most eyes that were analyzed, stable IOP reduction was observed in different types of pediatric glaucoma. The average LogMAR VA improved at the final observation in all groups of patients with pediatric glaucoma. Chan et al. reported that over 73% of affected eyes experienced VA worsening (16). Long-term visual functional outcomes of infants were better than those of newborns with PCG (37). Moreover, the improvement in the average VA was more significant in the JOAG group than in the PCG group at the final follow-up in this study. The results suggest that an effective reduction in IOP might not save VA for all patients. Therefore, amblyopia should be considered after glaucoma surgery in children. The 9.15% rate of reoperation was highest in patients with PCG in this study, which was lower than the 18% rate in a Würzburg clinical study (28). This may be due to the development of anti-glaucomatous medicines and surgeries. Effective treatment of pediatric glaucoma, preserving vision, and improving amblyopia remain challenging for all pediatric glaucoma therapeutics.

This study had several limitations. First, the retrospective nature of this study may have led to bias. Second, no widely established or standardized management protocol guidelines currently exist. Last, this study was a hospital-based data analysis of patients with severe pediatric glaucoma who underwent surgical treatment. Therefore, population-based epidemiological studies are needed.

In summary, the present study summarizes the characteristics, epidemiology, management, and outcomes of pediatric glaucoma patients. PCG is the most common type in central China. This finding may be related to ethnicity and possibly consanguine marriages. Traumatic glaucoma was the most common type of secondary glaucoma in this study and may be caused by low levels of economic development and child neglect in rural areas. Elevated IOP, poor vision, and eye pain were the most common signs and symptoms. Some pediatricians are familiar with the common symptoms and refer patients to an ophthalmologist when necessary. Trab and MAT combined with Trab were the most common interventions used in this study, and AGV was administered for multiple surgical failures or secondary pediatric glaucoma. Effective IOP control was observed in the PCG, JOAG, and secondary pediatric glaucoma groups; however, the improvement in average VA was larger in the JOAG group than in the PCG group at the final follow-up. Pediatric amblyopia might require full attention during the entire treatment, especially after anti-glaucoma surgery. Effective preventive measures and more public education on glaucoma prevention and the importance of early diagnosis and treatment are necessary. Expanded medical services and education in glaucoma screening in rural places and in at-risk populations are required.

## Data Availability Statement

The original contributions presented in the study are included in the article/supplementary material, further inquiries can be directed to the corresponding author.

## Ethics Statement

This retrospective study was approved by the Ethics Committee of Henan Eye Hospital, Henan Eye Institute, and Henan Provincial People's Hospital Human Research Ethics Committee [ethics number is HNEECKY-2022(08)]. Written informed consent to participate in this study was provided by the participants' legal guardian/next of kin.

## Author Contributions

QL, CL, XF, and WC performed the initial clinical database searches. QL, HL, XY, and YD performed the statistical analyses. QL produced the first draft of the manuscript, tables, and supervised the study. All authors contributed to study revision, edited the manuscript, reviewed the manuscript, and approved the submitted version.

## Funding

QL received support from Henan Medical Science Foundation of China (SBGJ2018083) and the Basic Research Project of Henan Provincial Eye Hospital (No. 21JCQN005).

## Conflict of Interest

The authors declare that the research was conducted in the absence of any commercial or financial relationships that could be construed as a potential conflict of interest.

## Publisher's Note

All claims expressed in this article are solely those of the authors and do not necessarily represent those of their affiliated organizations, or those of the publisher, the editors and the reviewers. Any product that may be evaluated in this article, or claim that may be made by its manufacturer, is not guaranteed or endorsed by the publisher.

## Abbreviations

AGV: Ahmed glaucoma valve
IOL: intraocular lens
IOP: intraocular pressure
JOAG: juvenile open-angle glaucoma
MAT: microcatheter-assisted 360° trabeculotomy
PCG: primary congenital glaucoma
Trab: trabeculotomy
VA: visual acuity.
